# Lessons learned: avoiding bias via multi-state analysis of patients’ trajectories in real-time

**DOI:** 10.3389/fmed.2024.1390549

**Published:** 2024-06-17

**Authors:** Elisabeth Lucke, Derek Hazard, Marlon Grodd, Susanne Weber, Martin Wolkewitz

**Affiliations:** Institute of Medical Biometry and Statistics, University Hospital Freiburg, Freiburg, Germany

**Keywords:** lessons learned, avoiding bias, pandemic preparedness, multi-state models, real time, analysis strategies

## Abstract

**Objectives:**

Many studies have attempted to determine the disease severity and patterns of COVID-19. However, at the beginning of the pandemic, the complex patients’ trajectories were only descriptively reported, and many analyses were worryingly prone to time-dependent-, selection-, and competing risk biases. Multi-state models avoid these biases by jointly analysing multiple clinical outcomes while taking into account their time dependency, including current cases, and modelling competing events. This paper uses a publicly available data set from the first wave in Israel as an example to demonstrate the benefits of analysing hospital data via multi-state methodology.

**Methods:**

We compared the outcome of the data analysis using multi-state models with the outcome obtained when various forms of bias are ignored. Furthermore, we used Cox regression to model the transitions among the states in a multi-state model. This allowed for the comparison of the covariates’ influence on transition rates between the two states. Lastly, we calculated expected lengths of stay and state probabilities based on the multi-state model and visualised it using stacked probability plots.

**Results:**

Compared to standard methods, multi-state models avoid many biases in the analysis of real-time disease developments. The utility of multi-state models is further highlighted through the use of stacked probability plots, which visualise the results. In addition, by stratification of disease patterns by subgroups and visualisation of the distribution of possible outcomes, these models bring the data into an interpretable form.

**Conclusion:**

To accurately guide the provision of medical resources, this paper recommends the real-time collection of hospital data and its analysis using multi-state models, as this method eliminates many potential biases. By applying multi-state models to real-time data, the gained knowledge allows rapid detection of altered disease courses when new variants arise, which is essential when informing medical and political decision-makers as well as the general population.

## 1 Introduction

### 1.1 Background

Having emerged in December of 2019, the SARS-CoV-2 virus has brought with it a variety of challenges. Due to its diverse clinical courses and surging waves of patients, it has impeded the provision of appropriate resources for hospitalised patients. At the beginning of the pandemic, studies attempting to understand the characteristics of COVID-19 suffered from severe time-related types of bias due to length bias, immortal-time bias, competing risk bias, and selection bias (1). For example, Zhou et al. carried out a study in Wuhan, China, in which 613 cases out of 813 were excluded because these patients had not yet experienced an outcome (2). Similarly, in Chen et al. 525 out of 799 patients were excluded from the analysis because they were still hospitalised (3). However, as Bajaj et al. argue, those in a moderate condition may stay in the hospital for longer than those in a poor condition, because the latter may succumb more quickly (4). At the same time, those in a good condition stay in the hospital for a shorter time than the ones in a moderate condition, as the former are likely to be discharged sooner. Thus, by ignoring all active cases, a selection bias arises, in which patients in a moderate condition are excluded, as their stay in the hospital is likely to be longest.

In addition, various studies suffered from competing risk bias. Competing risks refer to situations where an individual is subject to multiple possible events, and the occurrence of one event precludes the occurrence of the others (5). Assuming death is the event of interest, for example, the possibility of in-hospital death is eliminated when an individual is discharged from the hospital. Hence, being discharged is a competing event to dying in the hospital. In survival analysis, disregarding the presence of competing events can lead to a severe bias in the results.

To address the problem of poor data quality and biassed samples, this paper shows how statistical analyses can be used to avoid these biases in the context of the COVID-19 pandemic, following the example of Hazard et al. with multi-state models (6). Multi-state models entail defining certain states and the transitions among them. Multi-state models have the advantage that they are very flexible. For example, depending on the desired complexity, states can easily be consolidated. This increases the comprehensibility of the plots and at the same time can simplify the analysis (7).

### 1.2 Research in context

Multi-state models have been used in a variety of research contexts. For example, in modern ecology, it is used in capture-recapture experiments because the multi-state models allow for simple incorporation of temporal variation in the transition rates by modelling the rates as a parametric function over time (8). Furthermore, multi-state models are commonly used in cancer clinical trials, where patients usually experience multiple disease stages (9). The complex transitions between these stages can be comprehensively analysed using multi-state models. In addition, multi-state models can be used for predictions. More specifically in the context of hospital data, Roimi et al. used a multi-state model to predict individual patients’ hospital states based on their characteristics, such as age and gender. Furthermore, they also carried out analyses to predict the total hospital utilisation (10). Similarly, Keogh et al. also predicted the length of stay in hospital wards during the COVID-19 pandemic based on the patients’ characteristics. However, they extended their work by introducing the concept of “conditional expected length of stay,” which is defined as the expected length of stay in a certain state, conditional on the complete pathway taken through the states (11). In yet another paper, the multi-state model is analysed with parametric methods, which has the advantage that these parameters can be used to carry out simulations (12). The advantages and disadvantages of a variety of multi-state modelling approaches are reviewed in (13).

The implication of disregarding competing events in statistical analyses has been discussed frequently among the research community. In (14), McCaw et al. outline the problem of competing risks based on two papers: in (15), Beigel et al. carried out a clinical trial evaluating the effect of remdesivir versus a placebo in hospitalised COVID-19 patients. Similarly, Li et al. conducted a trial to detect the effect of convalescent plasma as compared to the effect of the standard of care on hospitalised COVID-19 patients (16). In both studies, death is a competing event. In addition, Wolkewitz et al. carried out an analysis to determine the impact of the duration of mechanical ventilation on the development of pneumonia while considering extubation as a competing event (17). An unbiased result could only be obtained when the competing event was accounted for. Supplementary Table 2 in (18) shows an overview of papers published in high-impact journals with a competing risk problem. In all cases, being discharged alive was the competing event that should have been accounted for in the analysis. Ignoring this competing event led to an overestimation of the cumulative incidence of the event of interest which, in the cases of the papers mentioned in Supplementary Table 2, was death or a composite outcome of intubation or death. Furthermore, a systematic literature review of observational studies that evaluated drug effectiveness in patients with COVID-19, carried out by Martinuka et al. (19), assessed the studies on three common methodological pitfalls in time-to-event analyses, one of them being competing risk bias. Their results showed that only one paper out of 11 accounted for the competing risk of being discharged alive by extending the follow-up period for discharged patients. All the others suffered from a competing risk bias. This highlights the scope of the problem.

Whilst it is evident that the topic of competing risk and selection bias as well as the use of multi-state models for the analysis of hospital data is not new to the research community, the topics are rarely taken into consideration by clinicians. Hence, this work aims to illustrate the biases that early COVID-19 analyses were subject to and provide a simple and easily applicable solution to overcoming these sources of bias by using the multi-state methodology. In addition, the paper aims to show how the continuous use of multi-state models in hospital data analysis facilitates hospital planning in disease outbreak scenarios by using comprehensive data visualisation techniques, thereby enhancing pandemic preparedness.

## 2 Methods

### 2.1 Data

The data used to demonstrate the advantages of multi-state models was collected in the form of a nationwide Israeli COVID-19 registry. It was previously used by Roimi et al., who conducted a multi-state analysis to predict hospital capacity utilisation in Israel. The data was collected in real-time and includes the day-to-day clinical course of patients hospitalised for at least 1 day between March 1st and May 2nd of 2020. It furthermore includes information on the patients’ age, sex, and initial admission date.

### 2.2 Multi-state models

In the model, four states are defined: moderate/severe (M/S), critical, discharge, and death. A patient starts in M/S or critical and can change between these two states an unlimited number of times before either dying in the hospital or being discharged. Death and discharge were defined as absorbent states, forbidding any transition away from these states. Supplementary Figure 1 illustrates the multi-state model. One important characteristic of multi-state models is that they allow the inclusion of competing events. In this model, discharge is the competing event of death. It is important to classify discharge as a competing event because being discharged alters the probability of death, i.e., persons discharged from the hospital are likely to be healthier and therefore have a lower probability of dying than those hospitalised (5). Disregarding this in the analysis would bias the results. Supplementary Table 1 shows example clinical courses of 3 patients through this multi-state model.

As von Cube et al. described, the analysis of multi-state models implies the calculation of transition probabilities and transition-specific hazard rates (20). In this paper, transition probabilities are calculated using transition hazards, which are defined as the instantaneous risk of moving between two states. Moreover, in our model, the calculation of the transition probabilities is dependent on all hazard rates of the transitions. Further mathematical details of multi-state models are explained in von Cube et al. (20) and Wolkewitz et al. (21).

### 2.3 Statistical analyses

**Figure 1:**
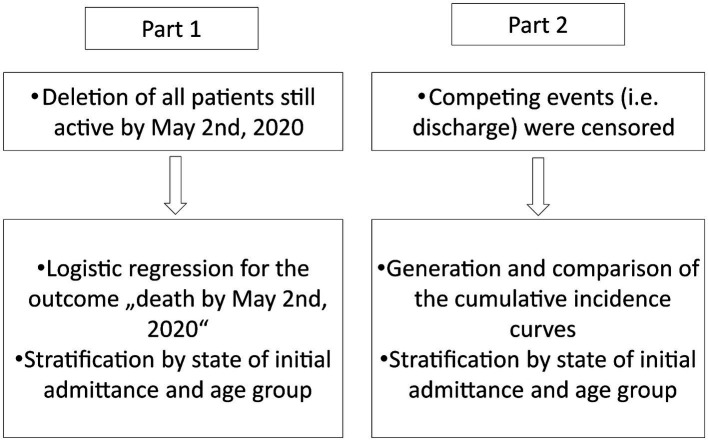
Methodology for the statistical analysis of the data set.

We used two different approaches to highlight the advantages of multi-state models over standard analysis techniques. In the first approach, we illustrated the bias which arises by excluding all active cases as was done in Zhou et al. and Chen et al. (2, 3). To do so, we excluded all active cases from our data and carried out a logistic regression for the outcome of “death.” Based on this regression, we predicted the probability for the event “death” to have occurred by May 2nd, 2020 for the different age groups. We stratified this analysis for the state at initial admittance. We then compared the results to the probability of dying using the multi-state model to highlight the discrepancies in the results if active cases are excluded. In the second approach, we demonstrated the bias that arises if competing events are censored. This means that only the event of interest, death, was considered. We created cumulative incidence curves for this model and compared them to the cumulative incidence curves obtained when considering the competing event of being discharged. This analysis was also stratified by age groups and the initial state of admittance. The methodology is displayed in Figure 1.

In addition, we analysed the multi-state model using stacked probability plots. Transition probabilities were calculated using the *mstate* package in R (Version 4.3.1) and the code created by Hazard et al. (6). First, cause/transition-specific Cox regressions were calculated. Cox models are a popular regression method in survival analysis. They are used when the effect of covariates on censored survival times is analysed. Cox regressions are calculated to compare how covariates affect the instantaneous risk of a transition between two states, i.e., the hazard ratio. For simplicity, the transitions M/S to critical and M/S to death were merged into one transition, to model how covariates affect the risk of clinical decline from the M/S state. Similarly, the transitions critical to M/S and critical to discharge were merged into one transition to model the effect of the covariates on an improvement from the critical state. To measure the effect of the month of admittance, a binary variable was used differentiating between admission in March and admission in April. The few cases that were admitted in May were included in April. After performing Cox regression, the baseline hazard was calculated, which is the hazard of the event of interest occurring at a certain point in time if the effect of all other covariates is zero. Then, the transition probabilities were calculated based on the baseline hazards. Finally, the transition probabilities were used to calculate the expected length of stay (ELOS) as shown in Hazard et al.

## 3 Results

The dataset included 2,675 patients of which 1,319 were younger than 60, 870 were between 60 and 80, and 486 were over the age of 80. 2,480 patients were originally admitted in an M/S state and only 195 patients were in a critical state at the time of admittance. By May 2nd, 2020, 198 patients had died and 311 patients (11.6%) were still active. Hence, by excluding all active cases, the data was reduced to 2,364 patients with 1,233 below 60, 734 between 60 and 80, and 397 over the age of 80. Of the 2,364 patients, 2,233 were initially in an M/S state and 131 in a critical state.

### 3.1 Bias in research during the COVID-19 pandemic

#### 3.1.1 Selection bias

**Figure 2:**
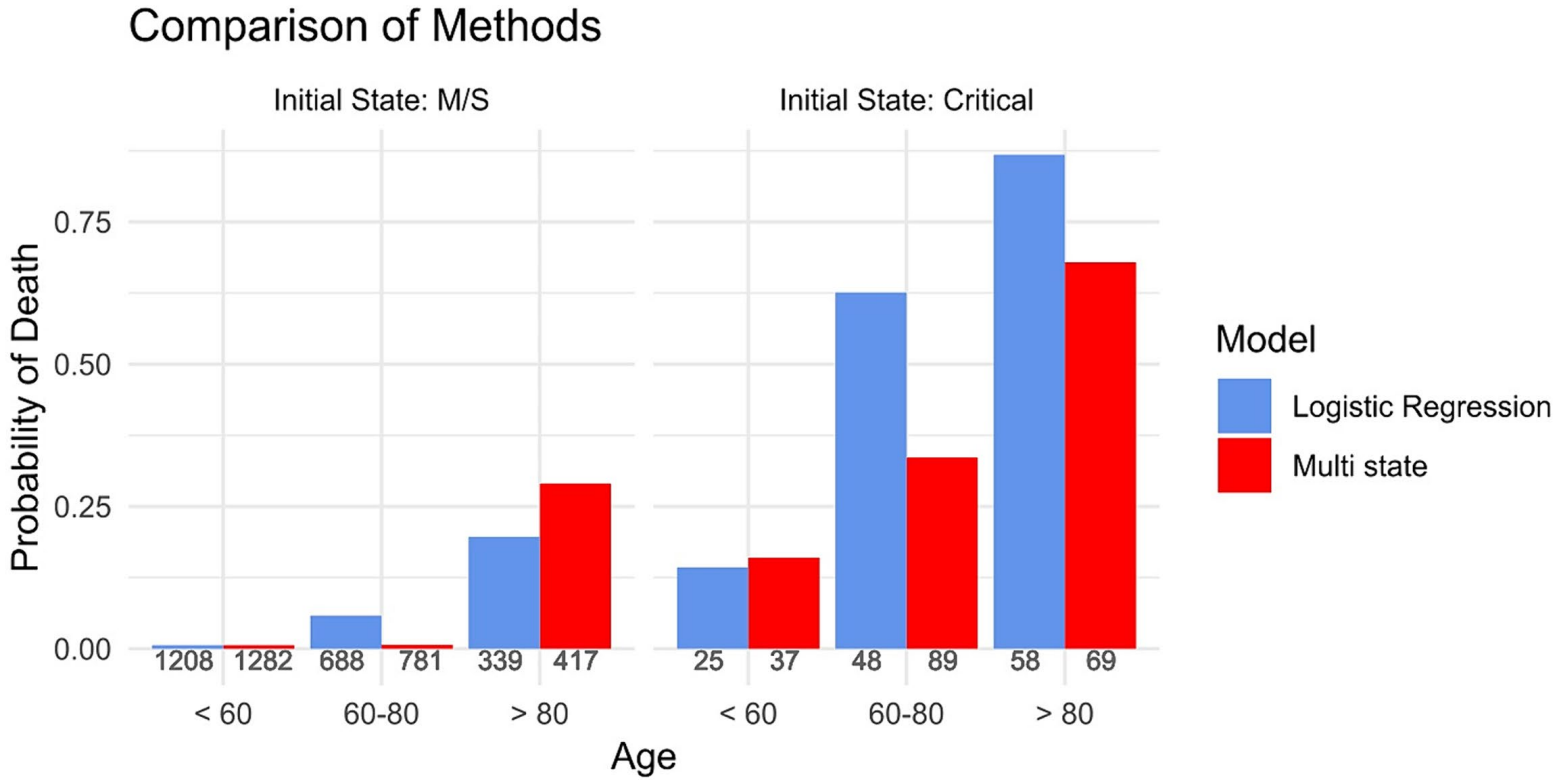
The probability of dying stratified by the initial state of admittance and age group, calculated once based on a subsample that excluded all active cases (Logistic Regression) and once based on the full cohort using multi-state methods (Multi-state). The numbers underneath each bar indicate the amount of people in the respective group.

Figure 2 depicts the predicted 30-day hospital mortality obtained using a logistic regression model when excluding all active cases from the data. As a comparison, it also shows the predicted 30-day hospital mortality obtained when the entire cohort was analysed using multi-state models. The graph shows that when excluding active cases from the analysis the probability of death is overestimated in the groups of patients where more deaths occurred, such as in the older age groups and in those who were initially admitted in a critical state. For example, when analysing the biassed cohort, the probability of death of the patients between 60 and 80 who were initially admitted in a critical state is 0.63. In comparison, when including all individuals initially admitted in a critical state, the probability of death is 0.34. The same pattern is seen for patients above 80 when initially admitted in a critical state. All numeric values of the 30-day hospital mortality can be found in Supplementary Table 6.

#### 3.1.2 Competing risk bias

**Figure 3:**
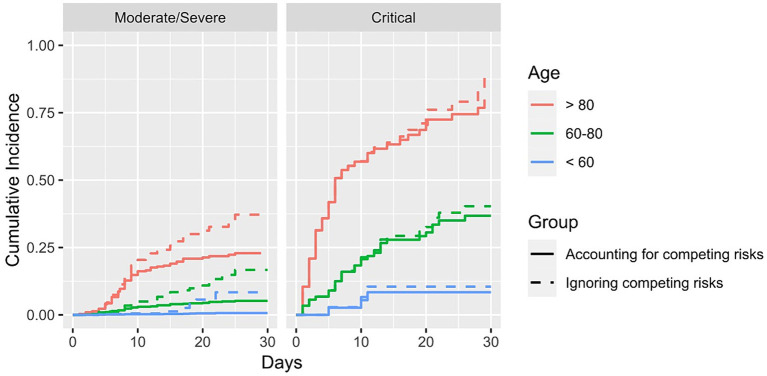
Cumulative incidence of dying stratified for age group and initial state of admittance. Solid lines show the results in which the competing risk of being discharged is accounted for, whereas the dashed lines show the results where the competing risk was not accounted for.

Figure 3 shows the cumulative incidence curves of two different models. Whilst the event of interest in both models was “death,” the event “discharged” was only classified as a competing event in one model (“Accounting for competing risks”). In the other, this competing event was ignored (“Ignoring competing risks”). The results show that especially for the patients who were initially admitted into a moderate/severe state, there are large discrepancies in the cumulative incidence curves between these two models. The method where competing risks are ignored overestimates the cumulative incidence of the event “death.” For example, the cumulative incidence of death after 30 days when ignoring the presence of competing risks is 0.37 whereas the cumulative incidence when considering the competing risk is 0.24.

### 3.2 Advantages of multi-state methods to avoid bias

Having demonstrated the bias that arises through standard methods of analysis that were used at the beginning of the COVID-19 pandemic, the following results highlight the advantages of using multi-state models.

#### 3.2.1 Planning bed capacity

**Figure 4:**
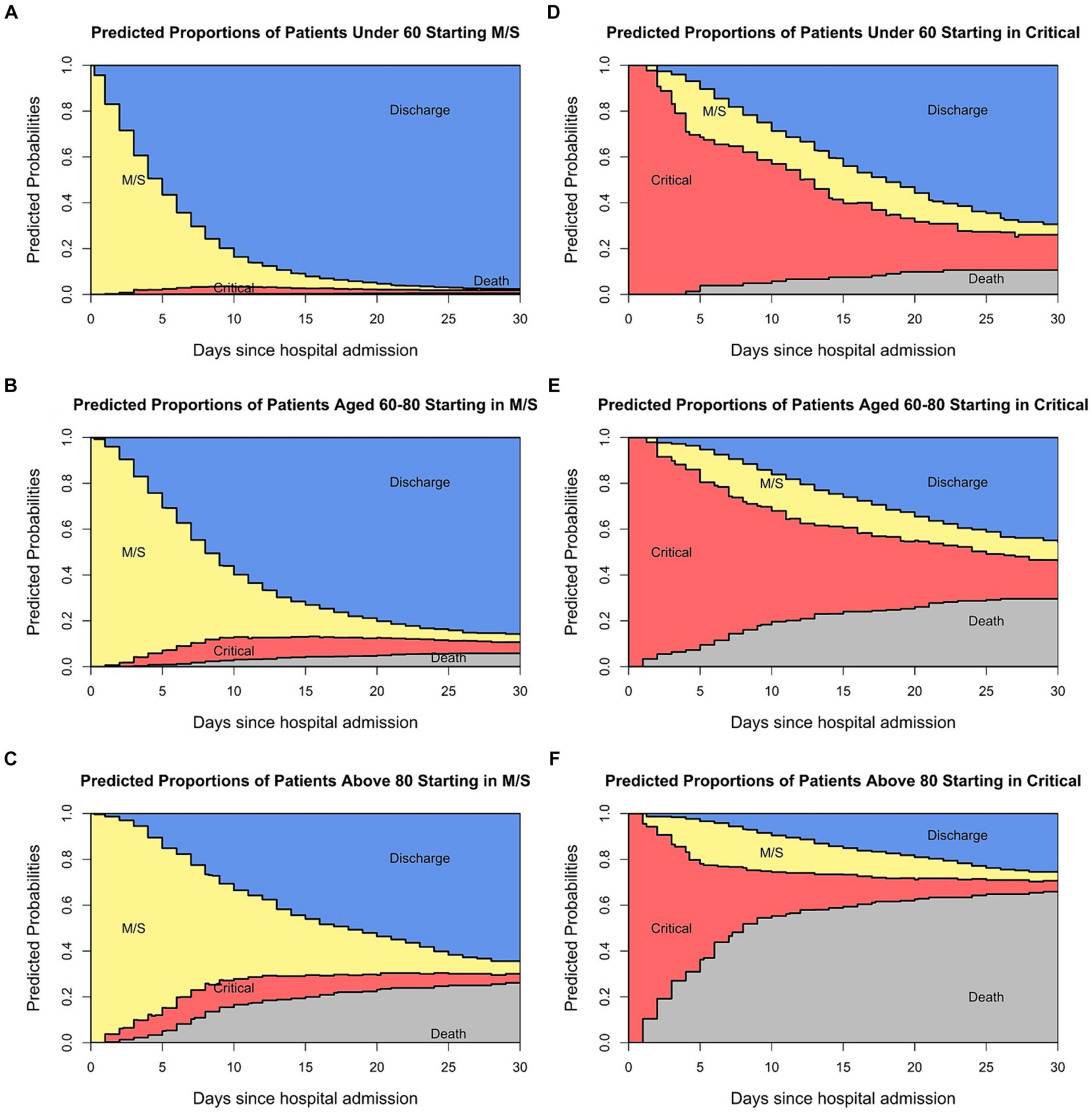
Predicted probabilities of being in each state at specific times for the patients admitted in a moderate state **(A–C)** and those admitted in a critical state **(D–F)**.

Figure 4 shows the estimated probabilities in each state over time stratified for age groups and initial state of admittance. The estimated probability of each state on any day can be determined. Figures 4A–C shows the predicted proportions of patients admitted in a moderate/severe condition in each state over time stratified by age group. For patients between 60 and 79, 30 days after hospitalisation 3.5% are in M/S, 4.7% are in critical, 85.9% are discharged and 5.9% are dead. This information is valuable for the standard care units. Similarly, Figures 4D–F shows the estimated probabilities in each state over time of those patients being admitted in a critical state. This information is relevant for the intensive care wards as it indicates how long patients stay in a particular condition and more importantly, in which condition the patients leave the ward. For example, for patients between 60 and 79, 30 days after hospitalisation 8.5% are in M/S, 16.4% are in critical, 45.5% are discharged, and 29.6% are dead. Thus, not only are these graphs useful when it comes to the planning of the different wards in the hospital, but by comparing the wards (standard care and ICU) with one another they can be useful in identifying disease patterns.

In addition, four different Cox regressions were constructed based on the model. As explained, for simplification purposes transitions one and three (as depicted in Supplementary Figure 1) were merged into one model as well as transitions four and five. The age group, sex, and initial admission date were included in the model as covariates. Overall, the age group and the binary covariate indicating the admission date (March versus April) were significant in most regressions. An increased age was associated with an increased hazard rate of transitions from M/S to critical or death, and from critical to death. The full result of the regressions is included in the Supplementary Tables 2–5 and it shows how multivariable Cox regressions can be used as an outlook to analyse how certain characteristics are risk factors for a specific transition.

Furthermore, to show how the multi-state models can be used to prepare healthcare providers for future COVID-19 waves, the ELOS in the two non-absorbent states was calculated as an example for patients between 60 and 80. For those admitted in a M/S condition, the ELOS in M/S is 9.28 days and the ELOS in the critical state is 2.75 days. In contrast, those admitted in a critical condition have an ELOS of 4.96 days in the M/S state and an ELOS of 15.91 days in the critical state, when estimating from the first day of hospitalisation. Supplementary Figure 2 shows the days in the M/S and critical state for each age group of the entire cohort.

#### 3.2.2 Analysis to study the most recent developments in real-time

**Figure 5:**
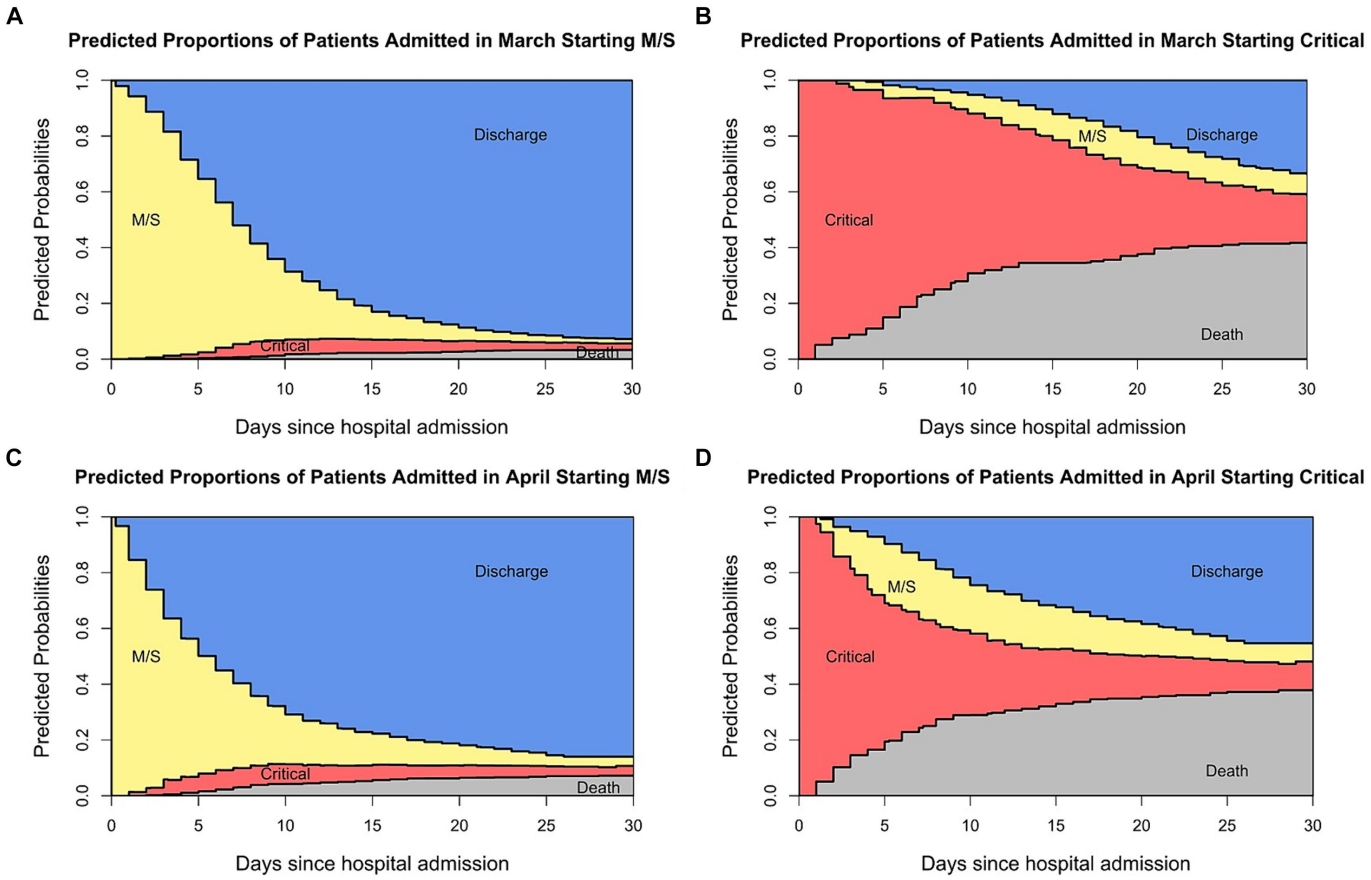
Stacked probability plots stratified by admittance date and initial state.

In addition, multi-state models allow the study of the most recent disease developments in real time. As an example, Figure 5 shows the stacked probability plots for the whole population stratified by the admittance date and initial admittance state. By stratifying for the hospital admission date, we show real-time changes in the clinical patterns of COVID-19. For those admitted in the M/S state, the mortality is higher when admitted in April/May than in March. This, however, is different when starting in the critical state. Here, the mortality seems to be lower for those admitted in April/May and the estimated probabilities over time in the critical condition are lower than for those admitted in March. Such information helps to clarify any observed differences in severity between the regular and the intensive care ward.

## 4 Discussion

In this paper, we compared standard survival analysis methods used for the analysis of COVID-19 hospital data in the beginning of the pandemic with advanced multi-state models. Supplementary Figure 2 and Supplementary Table 6 showed that selection bias led to an overestimation of death in the groups where many deaths occurred. As explained above, Bajaj et al. pointed out that excluding active cases biases the cohort towards the very ill and those that are only very lightly diseased. In our analysis, we manage to separate the severely ill from those that have a light course of the disease by stratifying for the initial state of admittance. Hereby, we show that our results support the claim of Bajaj et al., as Figure 2 shows that the probability of dying is overestimated in the patients admitted in a critical state, thus in the severely ill patients. These results are only partly reproducible in the opposite sense, i.e., showing that the probability of death is underestimated in the patients initially admitted in a M/S state. Whilst we see an underestimation for patients above 80 admitted in an M/S state, this cannot be observed for the other age groups. This could perhaps be explained by the small percentage of patients admitted in M/S that died, leading to an imprecise prediction of the probability of dying. In addition, Figure 3 shows that competing risk bias also leads to an overestimation of death because when ignoring competing risks, the model does not differentiate between being discharged and being hospitalised. Thus, those discharged are assumed to have the same risk of the event of interest as those who are still hospitalised. Consequently, the cumulative incidence is overestimated. In contrast, the cumulative incidence curves for the patients initially admitted in the critical state are very similar, as fewer patients are discharged. These results suggest that numerous analyses carried out at the beginning of the pandemic overestimated the severity of the disease. This is relevant as the overestimation may have led to some of the harsh public health measures, such as the closure of schools, which have, in retrospect, faced criticism for having been disproportionate (22). Hence, this example highlights the importance of obtaining unbiased information on disease severity, in which, as outlined above, multi-state models prove to be very useful.

In addition to highlighting the benefits of multi-state models in eliminating sources of bias, this paper also described further advantages of using multi-state models and corresponding stacked probability plots. One advantage is the potential these models have to assist the resource organisation of the hospital. By integrating stacked probability plots into the analysis, vital information on patients’ clinical courses over time can be displayed comprehensively and concisely. Furthermore, through the use of Cox regressions and the calculation of the ELOS, risk factors can be identified and the disease courses of individual patients can be predicted, thereby facilitating the planning of the hospital wards.

Additionally, stacked probability plots are easily interpretable and thereby facilitate communication with the general public. As van Schalkwyk et al. writes, the COVID-19 pandemic came at a time when distrust in institutions among the population was growing, for example through a change of government (23). This distrust worsened during the pandemic, as information from the media or research community was misunderstood by the population. However, stacked probability plots facilitate the interpretation of information conveyed by professionals. For example, Berger et al. carried out a country-level analysis of hospital capacity and utilisation. Besides measuring how different countries increased their ICUs as a response to COVID-19, they compared how long patients stayed in the ICU (24). However, the length of stay in the ICU can only be compared among countries if the mortality rate is the same. Otherwise, the comparison of ICU stations would not be meaningful because patients may leave the ICU due to death or due to discharge. The stacked probability plots manage to depict this idea by showing that the length of stay in the critical ward is determined by the occurrence of other states.

The utility of the plots to analyse real-time clinical patterns is especially highlighted in Figure 5, where the plots are stratified by admittance date. This is particularly clinically informative when new variants arise. Throughout the pandemic, various SARS-CoV-2 variants have emerged. The Delta variant was termed a variant of concern after its identification in India in May of 2021. It has increased transmissibility and virulence, as seen by elevated death and hospitalisation rates (25). However, there is no difference in characteristics between the wild-type virus and the Delta variant when compared by age and sex (26). In November of 2021, the Omicron variant was labelled a variant of concern. Whilst this variant showed a reduced severity overall, it led to increased hospitalizations in children under the age of 1 year (27). It is crucial to have this information in real-time because, based on such knowledge, policymakers could implement rules protecting small children and their parents, e.g., by allowing home office. Thus, it can be seen how multi-state models and stacked probability plots facilitate the communication of the disease and its real-time developments to the general public. This highlights that in the context of pandemic preparedness, it is imperative to collect high-quality hospital data continuously and promptly to understand the characteristics of the virus and to plan health care provision accordingly.

The strengths of this study are that it uses examples from the first pandemic wave to illustrate the extent to which the two forms of bias, selection bias, and competing risk bias, impact the results obtained from COVID-19 hospital data. Furthermore, the study provides an alternative approach that solves the shortfalls of standard methods and is therefore ideal for use in survival analysis in settings with more than one possible event. The limitation of the study is that it only includes data collected during the first pandemic wave. Our research would greatly benefit from analysing the clinical course of SARS-CoV-2 variants, as this would further illustrate the potential to detect differences in clinical characteristics using multi-state methods. However, whilst other data sets may exist of the time when variants were circulating, they could only be used if they are comparable to the Israeli data set of the first wave in terms of the characteristics of the population and the hospital service. Otherwise, false conclusions would be drawn. As such comparable data was unavailable, further variants were not included in the analysis.

## 5 Future works and conclusion

With enhanced data collection across Europe, future works could aim to demonstrate the effectiveness of multi-state models in detecting differences in clinical courses over longer periods. In addition, in future disease outbreak scenarios, e.g., influenza outbreaks, additional covariates can be incorporated into the analysis. These variables could aim at capturing differences in the risk profiles between the patients. Examples include demographic factors and health access disparities. Upon integration of such factors, the prediction of hospital capacity utilisation will become more accurate and thus, more personalised care can be provided to the patients.

In summary, this paper shows that in the context of pandemic preparedness, it is crucial to collect the right type of data to carry out appropriate, unbiased analyses, and thus aid efforts to overcome further pandemic waves. Hence, by showing the simple but detailed analyses that can be carried out with routine hospital registries as collected in Israel, this paper aims to improve the statistical analysis techniques used, thus obtaining unbiased information on the disease of interest in a timely manner so that public health measures can be implemented accordingly.

## Data availability statement

The original contributions presented in the study are included in the article/Supplementary material, further inquiries can be directed to the corresponding author.

## Ethics statement

Ethical approval was not required for the study involving humans in accordance with the local legislation and institutional requirements. Written informed consent to participate in this study was not required from the participants or the participants’ legal guardians/next of kin in accordance with the national legislation and the institutional requirements.

## Author contributions

EL: Conceptualization, Formal analysis, Methodology, Visualization, Writing – original draft. DH: Data curation, Formal analysis, Methodology, Software, Visualization, Writing – review & editing. MG: Conceptualization, Data curation, Supervision, Writing – review & editing. SW: Supervision, Writing – review & editing. MW: Conceptualization, Funding acquisition, Methodology, Resources, Supervision, Writing – review & editing.
