# Supplementary material for: Lessons learned: avoiding bias via multi-state analysis of patients’ trajectories in real-time
Source: Front Med (Lausanne). 2024 Jun 17;11:1390549. doi: 10.3389/fmed.2024.1390549 (PMC11215151; doi:10.3389/fmed.2024.1390549)
Supplement: Supplementary file 1 [file Data_Sheet_1.docx]

Supplementary Material

# Supplementary Figures and Tables

## Supplementary Figures


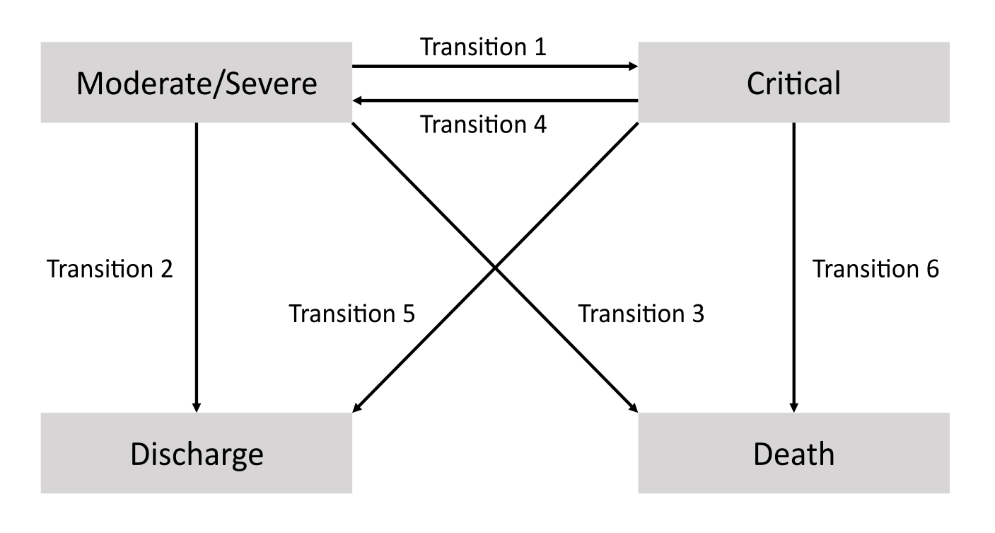


**Supplementary Figure 1.** Multi-state model with the 4 states moderate/severe, critical, discharge, death. The possible transitions between the states are numbered.


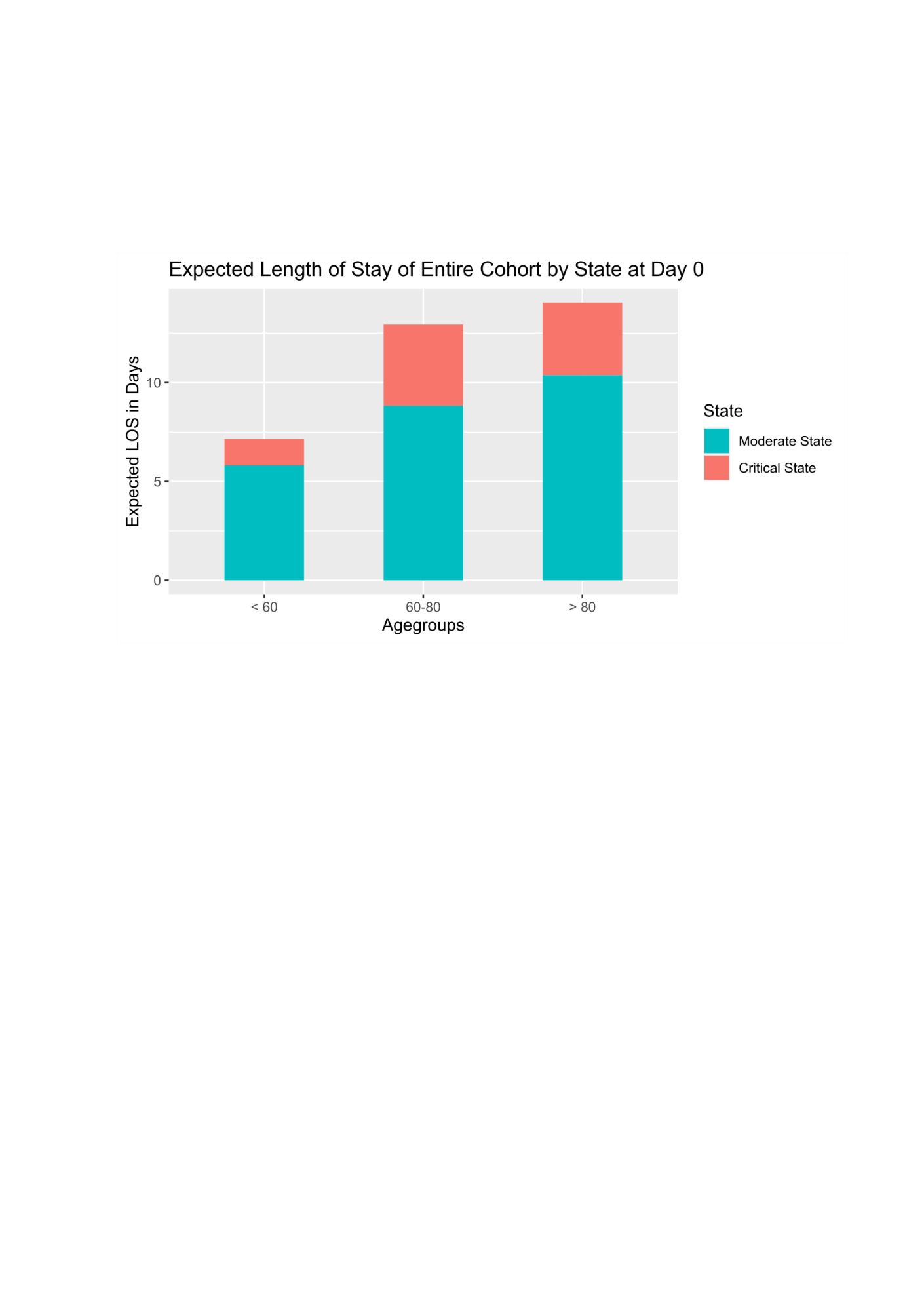


**Supplementary Figure 2.** The expected length of stay in each state stratified by age group at day zero.

## Supplementary Tables

**Supplementary Table 1.** Exemplary clinical course of patient 86, 87 and 109 with the days of entry and exit into each state. The patients’ sex, agegroup and respective transition type are also indicated. Half days (as for patient 86 and 109) were created when a patient transitioned twice within one day. For Example: Patient 87 was admitted to the hospital on day zero in a moderate/severe state. The patient stayed in that state until day three, on which the patient transitioned to the critical state. They stayed in the critical state for a day and finally passed away on day four.

| **ID** | **From** | **To** | **Entry** | **Exit** | **Sex** | **Agegroup** | **Transition** |
| --- | --- | --- | --- | --- | --- | --- | --- |
| 86 | 1 | 2 | 0.0 | 8.0 | Female | > 80 | 1 |
| 86 | 2 | 1 | 8.0 | 8.5 | Female | > 80 | 4 |
| 86 | 1 | cens | 8.5 | 21.0 | Female | > 80 | NA |
| 87 | 1 | 2 | 0.0 | 3.0 | Male | 60-80 | 1 |
| 87 | 2 | 4 | 3.0 | 4.0 | Male | 60-80 | 6 |
| 109 | 1 | 2 | 0.0 | 1.0 | Male | 60-80 | 1 |
| 109 | 2 | 1 | 1.0 | 1.5 | Male | 60-80 | 4 |
| 109 | 1 | 3 | 1.5 | 4.0 | Male | 60-80 | 2 |

**Supplementary Tables 2-5.** Cox regressions of the transitions.

| **M/S - Critical or Death** | | | |
| --- | --- | --- | --- |
| *Predictors* | *Estimates* | *CI* | *p* |
| sex [Male] | 1.45 | 1.16 - 1.79 | **0.001** |
| age > 80 | 3.70 | 2.74 - 5.00 | **<0.001** |
| age 60 - 80 | 2.30 | 1.74 - 3.05 | **<0.001** |
| date [April] | 1.99 | 1.59 - 2.50 | **<0.001** |

| **M/S - Discharge** | | | |
| --- | --- | --- | --- |
| *Predictors* | *Estimates* | *CI* | *p* |
| sex [Male] | 0.90 | 0.83 - 0.98 | **0.016** |
| age > 80 | 0.28 | 0.24 - 0.33 | **<0.001** |
| age 60 - 80 | 0.53 | 0.49 - 0.59 | **<0.001** |
| date [April] | 1.35 | 1.24 - 1.47 | **<0.001** |

| **Critical - M/S or Discharge** | | | |
| --- | --- | --- | --- |
| *Predictors* | *Estimates* | *CI* | *p* |
| sex [Male] | 0.58 | 0.44 – 0.76 | **<0.001** |
| age > 80 | 0.72 | 0.50 – 1.03 | 0.070 |
| age 60 - 80 | 0.77 | 0.57 – 1.03 | 0.081 |
| date [April] | 1.79 | 1.36 – 2.34 | **<0.001** |

| **Critical - Death** | | | |
| --- | --- | --- | --- |
| *Predictors* | *Estimates* | *CI* | *p* |
| sex [Male] | 0.71 | 0.51 - 1.01 | 0.054 |
| age > 80 | 7.13 | 3.76 - 13.52 | **<0.001** |
| age 60 - 80 | 2.33 | 1.21 - 4.46 | **0.011** |
| date [April] | 1.05 | 0.74 - 1.48 | 0.797 |

**Supplementary Table 6.** Comparison of 30-day hospital mortality obtained by logistic regression and obtained by multi-state models.

| **Initial state** | **Age group** | **Logistic regression** | **Multi-state models** |
| --- | --- | --- | --- |
|  | **< 60** | 0,006 | 0,006 |
| **Moderate/severe** | **60-80** | 0,058 | 0,007 |
|  | **> 80** | 0,197 | 0,290 |
|  | **< 60** | 0,143 | 0,160 |
| **Critical** | **60-80** | 0,626 | 0,336 |
|  | **> 80** | 0,868 | 0,679 |
